# Feasibility and structure of a state hospital botulinum neurotoxin clinic for clozapine-induced sialorrhea

**DOI:** 10.1186/s12991-025-00592-8

**Published:** 2025-08-26

**Authors:** Ai-Li W. Arias, Michael A. Cummings, George J. Proctor, Jonathan M. Meyer

**Affiliations:** 1 Department of Psychiatry and Neuroscience, University of California, Riverside, SOM Education Building, 900 University Avenue, Riverside, CA 92521 USA; 2 Department of Psychiatry & Human Behavior, Neuropsychiatric Center, University of California, UC Irvine Medical Center, 1010 The City Drive, Building 3, Route 88, Irvine, Orange, CA 92868 USA; 3 Psychopharmacology Resource Network, Clinical Operations Division, California Department of State Hospitals (DSH), Allenby Building, 1215 O Street, Sacramento, CA 95814 USA; 4 Department of Psychiatry, Loma Linda University School of Medicine, 11175 Campus Street, Loma Linda, CA 92350 USA; 5 Department of Psychiatry, University of California, 9500 Gilman Drive, San Diego, La Jolla, MC, CA 0603, 92093-0603 USA; 6 Department of State Hospitals, 3102 East Highland Avenue, Patton, Patton, CA 92369 USA

**Keywords:** Clozapine, Sialorrhea, Hypersalivation, Drooling, Botulinum neurotoxin, Botulinum toxin, Clinic

## Abstract

Clozapine is uniquely effective for treatment-resistant schizophrenia, treatment-resistant mania, and for aggression, suicide, or psychogenic polydipsia related to schizophrenia. Among its adverse effects, sialorrhea is an important barrier to clozapine treatment. Topical anticholinergic medications show limited efficacy, while systemic anticholinergics carry elevated risks of constipation, bowel impaction, or ileus. A superior treatment option for clozapine-induced sialorrhea exists in the form of periodic injection of major salivary glands with botulinum neurotoxin. This paper describes the feasibility and logistical issues involved in establishing a botulinum treatment clinic within a forensic psychiatric hospital. Critical elements in establishing a successful botulinum clinic for treatment of sialorrhea include adequate hospital administrative support, sufficient nursing staff, clinician injection training, education of treating psychiatrists regarding the availability and effectiveness of botulinum treatment for sialorrhea, and development of a clinic protocol, including procedural elements and relevant rating scales. Finally, botulinum treatment was evaluated to be cost-effective.

## Introduction

Clozapine is the only antipsychotic agent with demonstrated efficacy for treatment resistant schizophrenia, treatment resistant mania, and for nonpsychotic aggression, suicidality and primary polydipsia associated with schizophrenia spectrum disorders. However, clozapine possesses an array of unique adverse effects, especially sialorrhea, which itself affects at least 90% of clozapine-treated patients. Sialorrhea presents several concerns during clozapine treatment including patients’ desire to discontinue clozapine therapy, psychosocial stigmatization, and medical complications or morbidities (i.e., pneumonia related to nocturnal aspiration) [1, 2]. Clozapine and its active metabolite norclozapine possess muscarinic receptor agonist and antagonist properties, with the net clinical impact depending on the balance of these properties. Clozapine is predominately anticholinergic and is thought to be primarily responsible for inducing complaints of constipation, while the cause of sialorrhea is thought related to salivary gland M_1_ receptor agonism from norclozapine. As clozapine’s array of activities for treatment-resistant psychosis and aggression are especially valuable in forensic populations, the development of sialorrhea management protocols tailored to this population are of paramount importance in minimizing patient refusal and patient morbidity [3].

Given clozapine’s penchant for causing constipation, the preferred initial treatments for sialorrhea have been locally acting anticholinergic agents (e.g., sublingual atropine drops or intraoral ipratropium bromide spray) which have minimal systemic absorption. Usually, administration methods involve placing atropine drops sublingually or spraying ipratropium bromide onto the buccal mucosa, with the agent then held in the mouth for 15 and 30 s, respectively, prior to being rinsed out with circa 5 ml of water. When these topical agents fail, a systemic alternative such as glycopyrrolate can be used. The advantage glycopyrrolate has over centrally acting anticholinergics (e.g. benztropine or trihexyphenidyl) is that it does not cross the blood-brain barrier; however, the risk for potentially fatal ileus is doubled in comparison to no anticholinergic treatment or locally acting agents [4].

Prior to pursuing glycopyrrolate, and its risk for worsening constipation, there is another evidence-based alternative described in medical literature dating back two decades that involves botulinum toxin injected directly into salivary glands. In nature, there are several botulinum toxins (BT), all of which disable cholinergic neurotransmission by attacking the proteins involved in vesicle fusion in the presynaptic cholinergic neuron. There are 2 approved BT forms, A and B, which have slightly different activities; type A cleaves synaptosome-associated protein (SNAP-25), while type B targets vesicle-associated membrane protein (VAMP). Both types have traditionally been used to paralyze muscles in neurological conditions such as dystonia, for cosmetic purposes, or for treatment of migraine headaches. Starting in 2001, papers emerged on the use of BT to decrease salivary secretion in patients with neurological conditions marked by sialorrhea such as Parkinson’s disease (PD) or cerebral palsy (CP) [5]. Researchers subsequently examined BT use for clozapine-induced sialorrhea (CIS) with the hope of obviating the need to use systemic anticholinergics when locally applied agents are insufficiently effective [6].

Given elevated prevalence of treatment-resistant schizophrenia in state hospitals and forensic settings, and the widespread need to use clozapine for these patients, there is a compelling need to provide an evidence-based treatment for sialorrhea to avoid the significant concerns about constipation, ileus, and bowel obstruction when systemic anticholinergics are used to treat clozapine-induced sialorrhea [7, 8]. 

## Uses for botulinum toxin

**Fig. 1:**
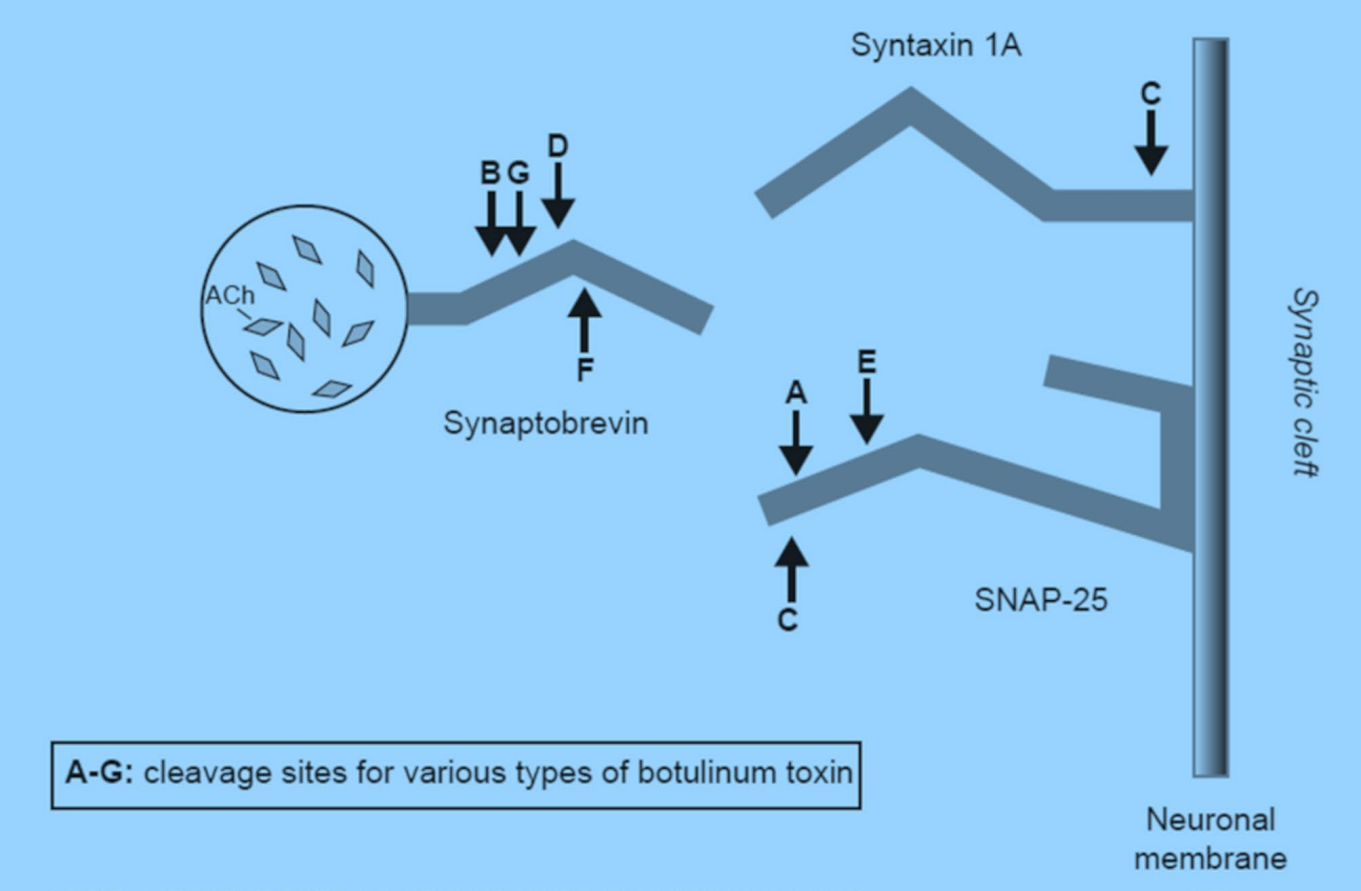
Target sites for BT types on SNARE proteins Adapted from Barr, et al. (2005). Botulinum neurotoxin detection and differentiation by mass spectrometry, *Emerging Infectious Diseases*, 11, 1578–1583 [20].)

Native BT consists of a 150 kD neurotoxin with a non-clostridial protein complex (e.g., hemagglutinins). There are 7 immunologically distinct serotypes which work via distinct mechanisms to inhibit the SNARE complex to cause a flaccid paralysis of target muscles in dystonia and for cosmetic applications. (See Fig. 1.) There are four type A toxins and one type B toxin currently approved in the U.S. Type A toxins include onabotulinumtoxina and abobotulinumtoxina (primarily used for cosmetic purposes and migraine headaches) [9], incobotulinumtoxinA (FDA-approved for blepharospasm, cervical dystonia, upper extremity spasticity, wrinkles, and sialorrhea), and prabotulinumtoxinA (used for glabellar wrinkles). RimabotulinumtoxinB is the type B toxin which is FDA-approved for cervical dystonia and for treatment of chronic sialorrhea. Although BT does not represent a cure for these conditions, it provides transient relief of symptoms lasting between 3 and 6 months [10–14].

The first studies documenting BT injection into salivary glands for Parkinsonian sialorrhea were published in 2001 based on the rationale that BT inhibits release of acetylcholine from synaptic vesicles of post-ganglionic parasympathetic fibers containing cholinergic receptors in the salivary glands [5, 15]. Further research noted that botulinum injections to the parotid and submandibular glands were easily administered and highly effective for 3 to 6 months when managing sialorrhea related to neurological conditions, and with no systemic effects. Benefits for medication-induced sialorrhea were subsequently documented [6, 16, 17].

## Botulinum neurotoxin for CIS

Although the 2 marketed forms of BT (A and B) possess slightly different mechanisms of action, but have substantial literature for sialorrhea in general, and significant case reports for CIS in particular. Due to the constant chemical stimulus from norclozapine’s M_1_ agonist properties, CIS may be greater in severity than other forms of sialorrhea, but the literature demonstrated that BT can reduce hypersalivation from all causes. Adverse effects from BT include pain or bruising at the site of inoculation, rare flu-like symptoms, headache, nausea, redness, dry mouth (in < 5% of cases), and unlikely possibility of temporary muscle paresis if toxin spreads beyond the injection area. Locally injected BT has virtually no risk of systemic adverse effects (e.g., breathing problems, trouble swallowing, muscle weakness, and slurred speech as observed in botulism).

In selecting the form of BT for CIS, we considered patient comfort during the injection, how discomfort might impact future acceptability of BT treatment, and issues of resistance to BT itself. Although rimabotulinumtoxinB is FDA approved for hypersalivation, there is literature which indicates greater pain with this injection compared to BT A [16]. For patients with severe mental illness who may be leery of a novel treatment that involves injections into the face, we opted for a toxin which imparts minimal pain on administration, incobotulinumtoxinA, administered with a 30-gauge needle. Primary resistance to actions for the toxin itself can appear following the first treatment and occurs at a rate of 6.25% in non-cosmetic applications [18, 19]. Primary resistance can arise if BT is used for conditions that respond only partially to BT (e.g., congenital ptosis, myasthenia gravis and some forms of spasmodic torticollis), when there is targeted muscle selection, and other treatment variables (e.g. high or too frequent dosing, improper storage or transport). In about 5–10% of non-cosmetic cases, individuals with previous success develop partial or no response to subsequent BT, treatments. This phenomenon, known as secondary resistance, is due to the development of antibodies to the toxin.

For these reasons and the novelty of this treatment approach in the state hospital system of California, we selected incobotulinumtoxinA based on its FDA approval for sialorrhea combined with lower risk for immunogenicity as evidenced by the fact that it is the only drug in its class not associated with the induction of secondary resistance [18, 19]. 

## Logistics for clinic set-up

Preparations for establishing a BT sialorrhea clinic involve the following, as noted in Table 1.

**Table 1:** Steps for Establishing a BT sialorrhea clinic

STEPS	DETAILS
***1***	**Training** • online training video by Merz Neurosciences indicating how to use landmarks to target the parotid and submandibular glands (http://www.xeomin.com/healthcare-professionals/indications/adult-chronic-sialorrhea/dosing/)[23] • one-day virtual or in-person botulinum toxin injection training using anatomical landmarks (sponsored by Merz Neurosciences)
***2***	**Creation of Clinical Forms** • referral form • informed consent form (ICF) • clinic protocol form with instructions for when medication is ordered by physician, proper labeling/storage of medication, supplies required, dosage chart, instructions for reconstitution of drug, and instructions for administration
***3***	**Choice of Efficacy Measures (see** Figs. 1 and 2**)** • Drooling Severity and Frequency Scale (DSFS) - completed by the treating physician for degree of saliva spillage and how much time during the day drooling occurs at various points in time during the initial treatment period beginning with the baseline visit and at weeks 4, 8, and 12 post-injection • Global Impression of Change Scale (GICS) − 7-point Likert scale that ranges from − 3 (very much worse) to + 3 (very much improved) to assess for treatment efficacy and response based on the subjective observations of both the patient and treating physician beginning 1 week after initial injection and at weeks 4, 8, and 12 post-injection
***4***	**Acquisition of Basic Supplies** • 1 ml 27–30 gauge (0.30–0.40 mm diameter), 12.5 mm length needle syringe • preservative-free 0.9% sodium chloride (injectable) • Xeomin^®^ (IncobotulinumtoxinA) 100-unit vial
***5***	**Clinic Personnel (minimum requirements)** • one physician – evaluates patient appropriateness for treatment, informed consent for the procedure, injection of incobotulinumtoxinA, assessing safety and efficacy, and determining dosing interval of maintenance injections • one nurse – reconstitute incobotulinumtoxinA with the 0.9% normal saline solution and procedure preparation
***6***	**Estimated Drug Cost (per Lexicomp)** • incobotulinumtoxinA = $613.20 per 100 U vial • estimated annual medication cost per patient (4 treatments/year) = $2,452.80

Training is critical for clinicians to map out landmarks and involves a brief video illustrating the anatomical landmarks (see Table 2), as well as a full-day live training with a neurologist experienced in this procedure. Although ultrasound guidance is not necessary for most cases, it could be considered in certain patients such as the elderly who might have gland atrophy. We recommend injecting parotid and submandibular glands bilaterally given the severity of CIS, but there is some debate on the issue [6, 17].

**Table 2:**
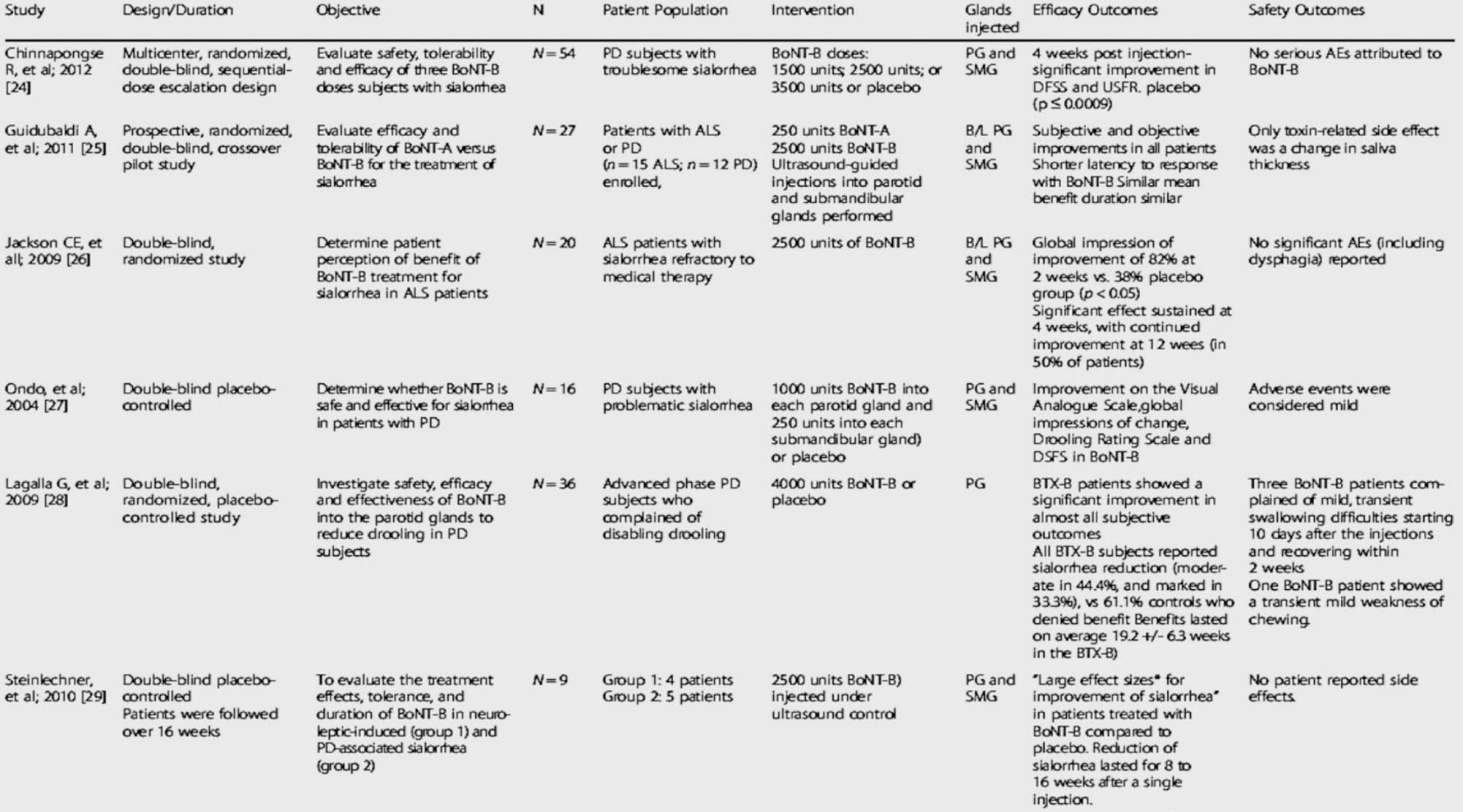
RimabotulinumtoxinB in sialorrhea: systematic review of clinical trials [17].

The paperwork (whether hardcopy or virtual) for such a clinic could be limited to three forms: referral, informed consent (ICF) and clinic protocol. Appropriate referrals for BT therapy for CIS include patients on clozapine and at least one anticholinergic concomitantly. The ICF is standardized to include what conditions BT is FDA-approved to treat, description of the procedure, risks/benefits, and signatures of the patient and administering physician. The clinic protocol includes the requisite basic supplies and instructions for handling and administering BT correctly.

**Fig. 2:**
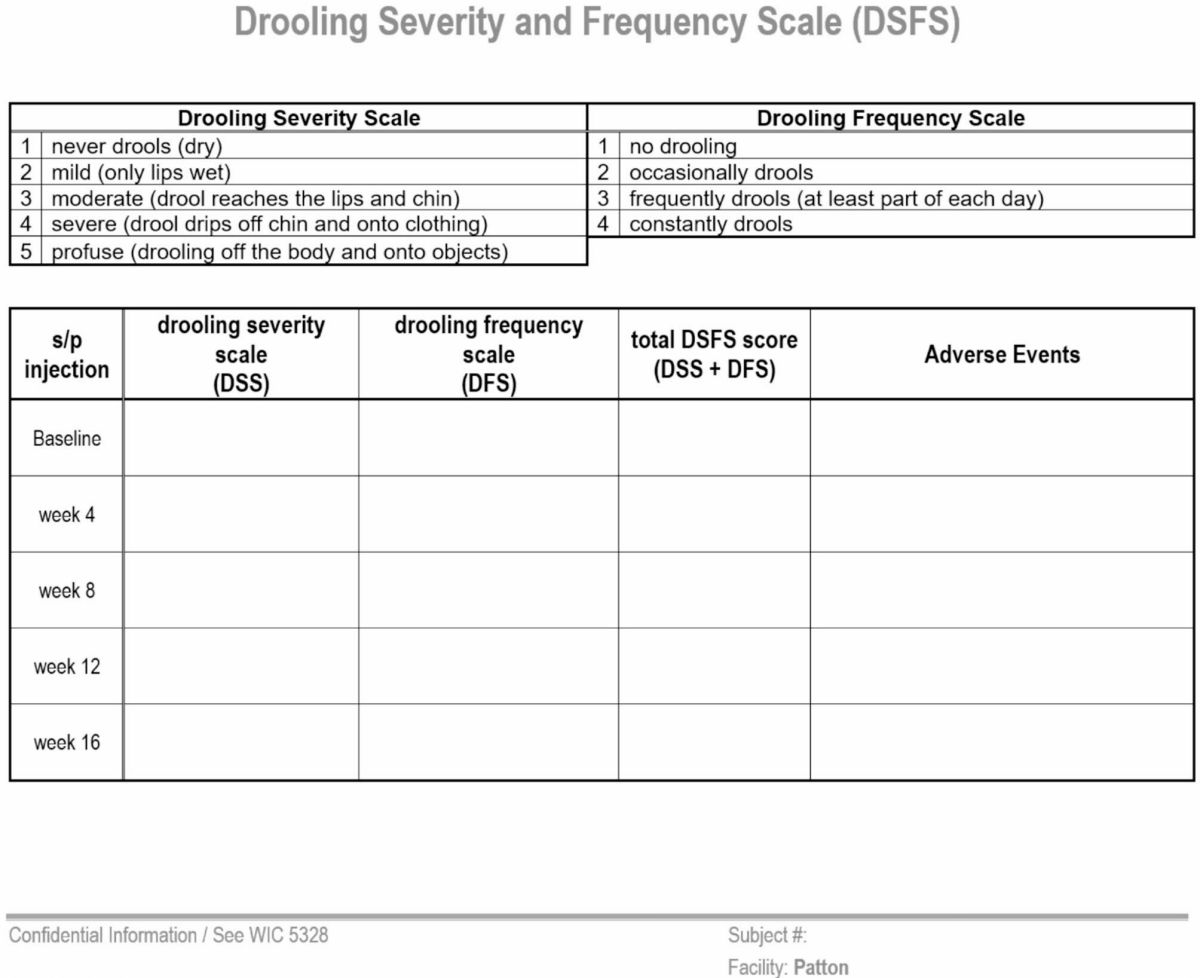
Drooling severity and frequency scale (Department of State Hostpials – Patton)

**Fig. 3:**
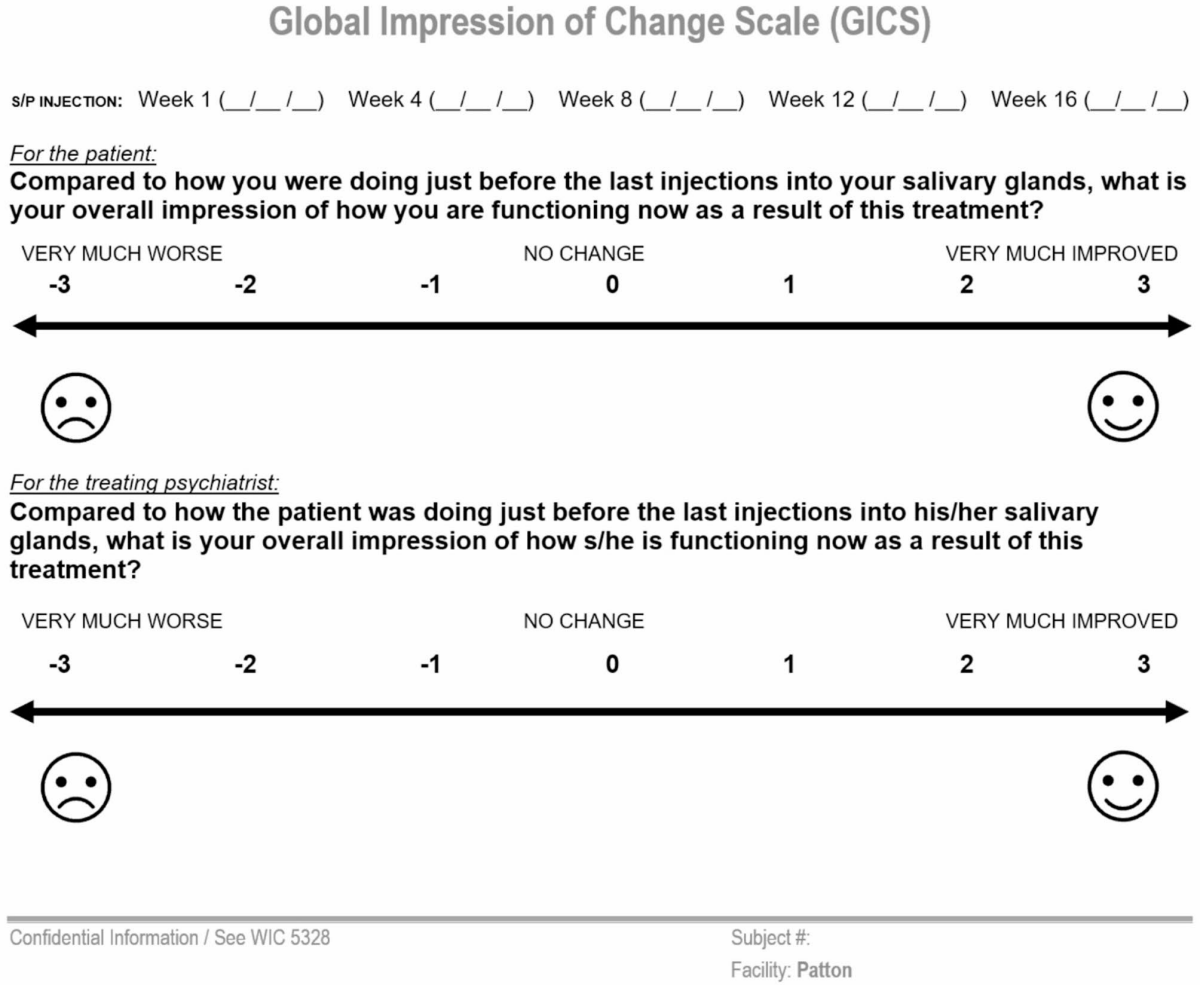
Global impression of change scale (Department of State Hostpials – Patton)

Objective and subjective efficacy measures can be used in the clinic. The objective measurement of unstimulated salivary flow rate (uSFR) using a digital scale calibrated to the thousandth of a gram and SalivaBio^®^ Oral Swabs takes approximately 1.25 h in the clinic; although important for clinical research, this is time consuming and not practical for routine treatment of CIS in a clinic. Subjective scales such as the Drooling Severity and Frequency Scale (DSFS) and Global Impression of Change Scale (GICS) are sufficient to determine efficacy of incobotulinumtoxinA for CIS [20, 21]. (See Figs. 2 and 3.)

With a nearly 90% efficacy rating, we anticipated a significant difference in the pre- and post-injection measurements, specifically a lowering of scores in the DSFS in the weeks following the injections [16]. 

When compared to other treatments provided to patients housed in a high security forensic setting, sialorrhea treatment with incobotulinumtoxinA is not unduly expensive As noted in Table 1, there are few specialized supplies necessary for clinic. Most outpatient clinics have, or can easily acquire, syringes with smaller gauge needles, straight forceps, rubbing alcohol, cotton/gauze pads and normal saline solution. The most significant supply cost is the medication itself, incobotulinumtoxinA. Based on U.S. wholesale pricing, the annual cost for anticholinergic agents used for sialorrhea approximates $704.10 annually, while incobotulinumtoxinA costs approximately $2,500 when dosed quarterly (Wholesale Acquisition Price = $613.20 per 100 U vial). If sialorrhea is inadequately controlled or systemic anticholinergic agents are used, the risk for aspiration pneumonia or ileus is elevated; pneumonia and constipation are the two most common reasons clozapine patients get hospitalized [22]. The annual cost of providing BT for CIS is significantly lower than the cost of acute hospitalization for one week in California ($27,853 with a daily rate of $3,979; figures based on information from the 2021 American Hospital Association Annual Survey in the Becker’s Hospital CFO Report).

## BoNT clinic protocol

In preparation for initial incobotulinumtoxinA injections, we asked treating psychiatrists to discontinue all systemic anticholinergics for sialorrhea one week prior to initial appointment to evaluate baseline sialorrhea severity; all anticoagulants and aspirin 5 days prior to appointment date; and all topical medications for sialorrhea 48 h before initial appointment to reduce injection site bleeding. Additionally, male patients were asked to shave the jawline area in the morning prior to the appointment time. After initial BoNT injections, patients resumed all anticoagulants and aspirin upon return to their home unit. Although improvement within the first week post-injection may be noticed, there may not be any changes for up to two weeks. Hence, pre-BoNT injection topical/systemic sialorrhea treatments were resumed but for 14 days only to mitigate risk for aspiration pneumonia during the lag period immediately following BoNT injections and then discontinued.

One hour before the patient gets to the clinic, the patient is asked to brush their teeth and remain NPO for at least one hour prior to the clinic appointment until after the 2 saliva samples are collected and injections administered. The patient is offered a drink of unflavored mineral water 30 min before the first measurement. Two swabs are placed between cheek and upper gums, and another two swabs are placed under the tongue medial to the lower jaw gum on each side of the patient’s mouth. These swabs remain in the patient’s closed mouth for 5 min, are removed using disposable forceps, and weighed on the calibrated scale. A half hour later, this process is repeated once more with no additional mineral water offered. After the average of the two uSFR measurements are obtained, the physician injector injects 30 U into each parotid gland and 20 U into each submandibular gland for a total of 100 U incobotulinumtoxinA per treatment. After the administration of incobotulinumtoxinA, the patients are permitted to eat and drink as usual. Follow-up visits for uSFR measurements are scheduled in 4-week intervals through 16 weeks post-injection during the initial treatment period with the first maintenance injection visit scheduled for 12 weeks after initial injections. We are measuring uSFR monthly for the first four months of treatment in order to determine safety and efficacy of botulinum toxin for drug-induced, resistant sialorrhea. However, quarterly uSFR measurements taken during the first year of treatment and then annually thereafter would suffice for a viable BoNT Clinic with no research goals.

## Discussion

Presented here is the first systematic protocol designed for the use of BT for CIS in a state hospital population. Important considerations included the compelling need to offer this treatment to avoid the complications of systemic anticholinergic agents when locally applied agents are ineffective and to utilize personnel comfortable with that population (e.g., staff psychiatrists and neurologists) to provide the injections on-site, thereby obviating need for transport to outside clinic, etc.

The experience at the California Department of State Hospitals shows that staff psychiatrists or neurologists could be trained in one day to deliver the injections and educating the referring clinicians on the protocol for stopping topical/systemic anticholinergic agents.

**Cost Analysis.** When compared to other treatments provided to patients housed in a high security forensic setting, sialorrhea treatment with incobotulinumtoxinA is cost-effective. As noted above, there is little by way of specialized supplies necessary for a BoNT clinic. Most outpatient clinics have, or can easily acquire, syringes with smaller gauge needles, straight forceps, rubbing alcohol, cotton/gauze pads and normal saline solution. The most significant supply cost for this type of sialorrhea treatment clinic is the medication itself, incobotulinumtoxinA. Based on the California Department of General Services Wholesale Acquisition Cost, the annual cost for anticholinergic agents used for sialorrhea is $704.10 while incobotulinumtoxinA costs approximately $2500 when dosed quarterly (Wholesale Acquisition Price = $613.20 per 100 U vial). Although the annual cost of incobotulinumtoxinA for sialorrhea is 3 to 4 times the cost of anticholinergic agents (10% efficacy), it is on the order of 9 times more effective. Minimal staffing needs for a BoNT Clinic is one physician and one nurse as described above.

In the initial months of this clinic using BT for CIS, it was clear that much emphasis needed to be placed on educating treating clinicians on following the treatment protocol, including educating their patients on what to expect and discontinuing anticholinergics two weeks post-injection.

Initial experience showed that all patients except for one were appropriately educated about the procedure, went through with the procedure, and tolerated it well. Collection of sialorrhea treatment response data was disrupted by the Covid-19 pandemic and a consequent shortage of clinic nursing staff. This one patient had no idea why they were at the clinic and balked at the idea of getting injections in the face. None of the patients who underwent this procedure reported any discomfort from the injections; all injections performed to date have been well tolerated without any complications. Preliminary efficacy data have yet to be analyzed but appear to be promising.

## Take-home points

- Local treatments are the preferred option for managing CIS, but many patients respond inadequately. BT offers evidence-based method to avoid systemic anticholinergic burden of agents such as glycopyrrolate.
- Physicians can be easily trained to administer these injections. No severe complications, such as injury to facial nerve, have been observed.
- Given the severity of CIS, bilateral injection into the parotid and submandibular glands appears indicated and is safe with no patients reporting xerostomia.
- All but one of 33 patients who receive this treatment tolerated the injections well with no adverse effects; the one patient only had transient perioral swelling.

## Data Availability

No datasets were generated or analysed during the current study.
